# Clinical pharmacist-led interventions and their impact on outcomes in patients with bipolar I disorder: a systematic review and meta-analysis

**DOI:** 10.3389/fmed.2025.1725491

**Published:** 2025-12-12

**Authors:** Moteb Khobrani, Sultan M. Alshahrani

**Affiliations:** Department of Clinical Pharmacy, College of Pharmacy, King Khalid University, Abha, Saudi Arabia

**Keywords:** bipolar I disorder, clinical pharmacist interventions, medication adherence, relapse prevention, quality of life, collaborative care, hospital readmissions

## Abstract

**Background:**

Pharmacist-led interventions have increasingly been recognized as effective strategies for improving outcomes in psychiatric care, yet their role in patients with bipolar I disorder (BD-I) remains underexplored. This systematic review and meta-analysis synthesized existing evidence on the impact of clinical pharmacist-led interventions (defined as medication education, medication review, adherence support, relapse monitoring, and collaborative care with psychiatrists) on medication adherence, relapse prevention, hospitalization, and quality of life among individuals with BD-I.

**Objectives:**

To evaluate the effectiveness of pharmacist-led interventions, either alone or in collaboration with psychiatrists, on key clinical and humanistic outcomes in adults diagnosed with BD-I.

**Methods:**

Electronic databases (PubMed/MEDLINE, Embase, PsycINFO, Scopus, Web of Science, and the Cochrane Library) were searched from January 2000 to August 2025 for randomized controlled trials and non-randomized quasi-experimental designs (prospective cohort studies and pre–post intervention studies) assessing pharmacist-led interventions in BD-I. Studies were screened and appraised independently in accordance with PRISMA 2020 guidelines, using the Cochrane RoB 2 tool for randomized controlled trials and the Newcastle–Ottawa Scale for non-randomized studies. Meta-analyses were performed using Review Manager (RevMan 5.4). Primary outcomes were medication adherence, relapse, hospitalization, and health-related quality of life (QoL). Secondary outcomes included any additional clinical or humanistic measures reported by the included studies. Effect sizes were expressed as odds ratios (ORs) for dichotomous outcomes and as mean differences (MDs) or standardized mean differences (SMDs) for continuous outcomes, each reported with 95% confidence intervals (CIs).

**Results:**

Five studies met the eligibility criteria, and four contributed quantitative data. Pharmacist-led interventions significantly improved medication adherence compared with usual care (MD = 1.47 [95% CI: 1.35–1.59], *p* < 0.00001). For relapse prevention, pooled analysis from two trials showed reduced relapse episodes (OR = 1.94 [95% CI: 1.15–3.23], *p* = 0.01; I^2^ = 38%). Improvements were also observed in quality of life (SMD = –1.89 [95% CI: –4.95–1.20], *p* = 0.21; I^2^ = 90%). Evidence for hospitalization reduction was limited but directionally favored pharmacist involvement.

**Conclusion:**

Clinical pharmacist-led interventions significantly enhance medication adherence and quality of life and may reduce relapse risk among patients with BD-I. However, given the limited number of studies and small pooled sample sizes, the overall evidence remains preliminary, and the findings should be interpreted cautiously rather than as definitive conclusions. Further large-scale, multicenter studies are warranted to confirm these benefits and assess cost-effectiveness.

**Systematic review registration:**

[https://www.crd.york.ac.uk/prospero/], identifier [CRD420251123737 04/10/2025].

## Introduction

Bipolar disorder (BD) is a chronic and recurrent psychiatric condition characterized by alternating episodes of mania, hypomania, and depression, interspersed with periods of euthymia (1). These mood fluctuations are associated with changes in energy levels, behavior, and cognitive functioning, often leading to substantial impairment in quality of life, occupational functioning, and interpersonal relationships (1, 2). Globally, BD contributes significantly to disability-adjusted life years (DALYs), reflecting both its high prevalence and severe impact on patients and healthcare systems (2, 3).

Among its subtypes, bipolar I disorder (BD-I) is distinguished by the presence of at least one manic episode, typically requiring clinical intervention or hospitalization (1). Despite the availability of evidence-based pharmacological and psychological treatments, adherence to long-term therapy remains suboptimal, increasing the risk of relapse, rehospitalization, and healthcare costs (4–7).

Medication adherence in BD has been widely studied, with systematic reviews showing persistent challenges across both bipolar and schizophrenia populations (4, 5). Commonly cited barriers include adverse effects, poor illness insight, and complex treatment regimens (6, 7). While expert consensus guidelines recommend multifaceted interventions to address adherence problems in serious mental illness (8), relatively few studies have examined tailored strategies specifically for BD-I.

Pharmacists, as accessible healthcare providers, are increasingly recognized for their role in supporting patients with mental illness through medication management, counseling, and collaborative care models (9–12). Previous systematic reviews have demonstrated the positive impact of pharmacist-led services in mental health broadly, including improvements in medication use, adherence, and continuity of care (9, 13, 14). However, evidence focusing exclusively on BD-I remains limited. Existing studies suggest potential benefits of pharmacist–psychiatrist collaborative education and long-term pharmacist interventions in bipolar disorder (15, 16). Yet, no prior systematic review and meta-analysis has synthesized the effectiveness of these interventions specifically for BD-I (13–16). Recent literature further emphasizes the expanding roles of clinical pharmacists in mental health settings, including comprehensive mental health pharmacy services across Europe (17), antipsychotic optimization in elderly patients with complex polypharmacy (18), and pharmacist-led medication reconciliation during transitions of care in psychiatric hospitals (19). Additional emerging evidence highlights the contribution of clinical pharmacists within daily interdisciplinary ward rounds in psychiatric facilities (20) and the importance of structured medication reviews for optimizing treatment among older inpatients with mental disorders and somatic comorbidities (21).

Given these expanding clinical roles, synthesizing the specific impact of pharmacist-led interventions in BD-I is essential to clarify their contribution to patient-centered outcomes and to inform the development of targeted mental health pharmacy services tailored to this population.

This systematic review and meta-analysis, therefore, aims to evaluate the impact of clinical pharmacist-led interventions on treatment outcomes in patients with BD-I, focusing on medication adherence, relapse prevention, hospitalization, and quality of life.

## Methods

### Study design

This study is a systematic review and meta-analysis conducted in accordance with the Preferred Reporting Items for Systematic Reviews and Meta-Analyses (PRISMA) 2020 guidelines (22). The protocol was prospectively registered in the PROSPERO international prospective register of systematic reviews (Registration ID: CRD420251123737).

### Eligibility criteria

Studies were eligible if they specifically enrolled adult patients (≥ 18 years) with a confirmed diagnosis of bipolar I disorder (BD-I) based on standardized diagnostic criteria such as the Diagnostic and Statistical Manual of Mental Disorders (DSM) or the International Classification of Diseases (ICD). Included studies evaluated clinical pharmacist-led interventions, either delivered independently or in collaboration with psychiatrists or other healthcare professionals, and compared these with usual care or non-pharmacist-led interventions. The primary outcome of interest was medication adherence, while secondary outcomes included relapse prevention, hospitalization rates, and health-related quality of life (QoL). These outcomes were selected based on their clinical relevance in BD-I management and their consistent reporting across eligible studies. Eligible designs included randomized controlled trials (RCTs), quasi-experimental, or prospective cohort studies, including pre–post intervention designs where baseline and follow-up quantitative data were available, provided that sufficient quantitative outcome data were available for extraction. Only peer-reviewed articles published in English were considered. No restrictions were placed on study duration or length of follow-up.

We excluded studies that focused primarily on other psychiatric disorders (e.g., schizophrenia or major depressive disorder) unless outcomes for BD-I were reported separately. Case reports, case series, conference abstracts, editorials, commentaries, and review articles were also excluded.

These eligibility criteria were established to ensure that only high-quality, clinically relevant evidence specific to BD-I was synthesized, thereby enhancing the validity and applicability of the findings to this patient population.

### Information sources and search strategy

A comprehensive literature search was conducted to identify relevant studies evaluating pharmacist-led interventions in patients with bipolar I disorder (BD-I). The following electronic databases were searched: PubMed/MEDLINE, Embase, PsycINFO, Scopus, Web of Science, and the Cochrane Library. In addition, trial registries (e.g., ClinicalTrials.gov and the WHO International Clinical Trials Registry Platform) and gray literature sources (e.g., conference proceedings and dissertations) were screened to minimize publication bias. Gray literature screening included manual searches of conference abstracts, institutional repositories, and dissertation databases to identify unpublished or non-indexed studies. The search encompassed all studies published in the database from January 2000 to August 2025, with no geographical restrictions. The year 2000 was selected as the lower limit because structured pharmacist-led mental health services and BD-specific collaborative care models began to emerge in the early 2000’s, and earlier literature provided limited or no relevant intervention data. The search approach combined free-text terms associated with bipolar I disorder and pharmacist-led interventions with controlled vocabulary (e.g., MeSH terms, Emtree). The search strategy was developed by combining BD-I–related terms with pharmacist-related keywords (e.g., pharmacist, clinical pharmacist, pharmacist-led, pharmacist intervention) and outcome terms related to medication adherence, relapse, and quality of life, applying Boolean operators (AND/OR) and controlled vocabulary (MeSH, Emtree) to match each database. Additional relevant systematic reviews and meta-analyses were also screened to ensure that no eligible primary studies were missed. Only studies specifically reporting outcomes for BD-I were included to maintain a focused and clinically homogeneous population for analysis (Supplementary material).

### Study selection

All records identified through the search were imported into reference management software, and duplicates were removed. Two reviewers (M.K. and S.M.A.) independently screened the titles and abstracts of all retrieved articles. Full texts of potentially relevant studies were then assessed in detail against the predefined eligibility criteria. Any disagreements between the two reviewers were resolved through discussion and mutual consensus, and no third reviewer was required. The study selection process adhered to the PRISMA 2020 guidelines.

### Data extraction and management

Data extraction and management were conducted systematically to ensure consistency and accuracy. Initially, EndNote X9 (Clarivate Analytics) was used to manage all records in order to find and eliminate duplicates. Following that, study selection and screening were carried out in Rayyan QCRI, a web-based platform developed by the Qatar Computing Research Institute to support independent and blinded screening in systematic reviews, which allowed for independent and blinded screening by two reviewers (23).

Before being fully implemented, a standardized data collection form was created in Microsoft Excel and tested on a small number of studies for data extraction. The following information was extracted from each included study: author (year and country), study objective, study design, sample size (N), setting, intervention (intervention group, IG), comparator or control group (CG), follow-up duration, and outcomes assessed (Supplementary material). Data were extracted independently by two reviewers, and any discrepancies were resolved through discussion until consensus was reached.

### Quality assessment

The methodological quality and risk of bias of the included studies were assessed independently by two reviewers. The Cochrane Risk of Bias 2.0 tool (RoB 2.0) was used for randomized controlled trials (RCTs). RoB 2.0 assesses five important areas: the randomization process, deviations from intended interventions, missing outcome data, outcome measurement, and selection of reported results. It generates an overall risk-of-bias judgment based on these criteria (24). For non-randomized and observational studies, the Newcastle–Ottawa Scale (NOS) was used to evaluate study quality in three areas for observational and non-randomized studies: outcome/exposure assessment, comparability of study groups, and participant selection (25).

### Data synthesis and statistical analysis

Depending on the accessibility and comparability of outcome measures, both narrative and quantitative methods were used to synthesize the data. Narrative synthesis involved summarizing key study characteristics, intervention components, and the direction of effects in instances where outcomes were heterogeneous or insufficient for pooling. For studies reporting sufficiently homogenous outcomes, meta-analysis was performed using a random-effects model to account for potential between-study variability, in line with recommendations from the Cochrane Handbook for Systematic Reviews of Interventions (26).

Continuous outcomes were summarized using mean differences (MDs) or standardized mean differences (SMDs) depending on the scale used, whereas dichotomous outcomes were presented as odds ratios (ORs) with 95% CIs. The I^2^ statistic was used to determine statistical heterogeneity, with values above 50% indicating substantial heterogeneity, and the chi-square test was used to evaluate it (*p* < 0.10 indicating significance) (22, 26). Findings were narratively synthesized in cases where meta-analysis was not practical.

All statistical analyses were conducted using Review Manager (RevMan, version 5.4, The Cochrane Collaboration) and Stata (version 17.0, StataCorp, College Station, TX, United States), with statistical significance set at *p* < 0.05 (two-tailed). Sensitivity analysis was prespecified to assess the robustness of pooled estimates by examining the impact of excluding the long-term follow-up extension study to avoid duplication of participant data.

### Subgroup analyses

Where sufficient data were available, subgroup analyses were planned to explore potential sources of heterogeneity, including intervention type (e.g., education-focused vs. medication management), duration of follow-up, and study setting. These analyses were intended to help identify whether specific intervention characteristics or study features influenced the magnitude or direction of treatment effects.

## Results

### Study selection

**FIGURE 1:**
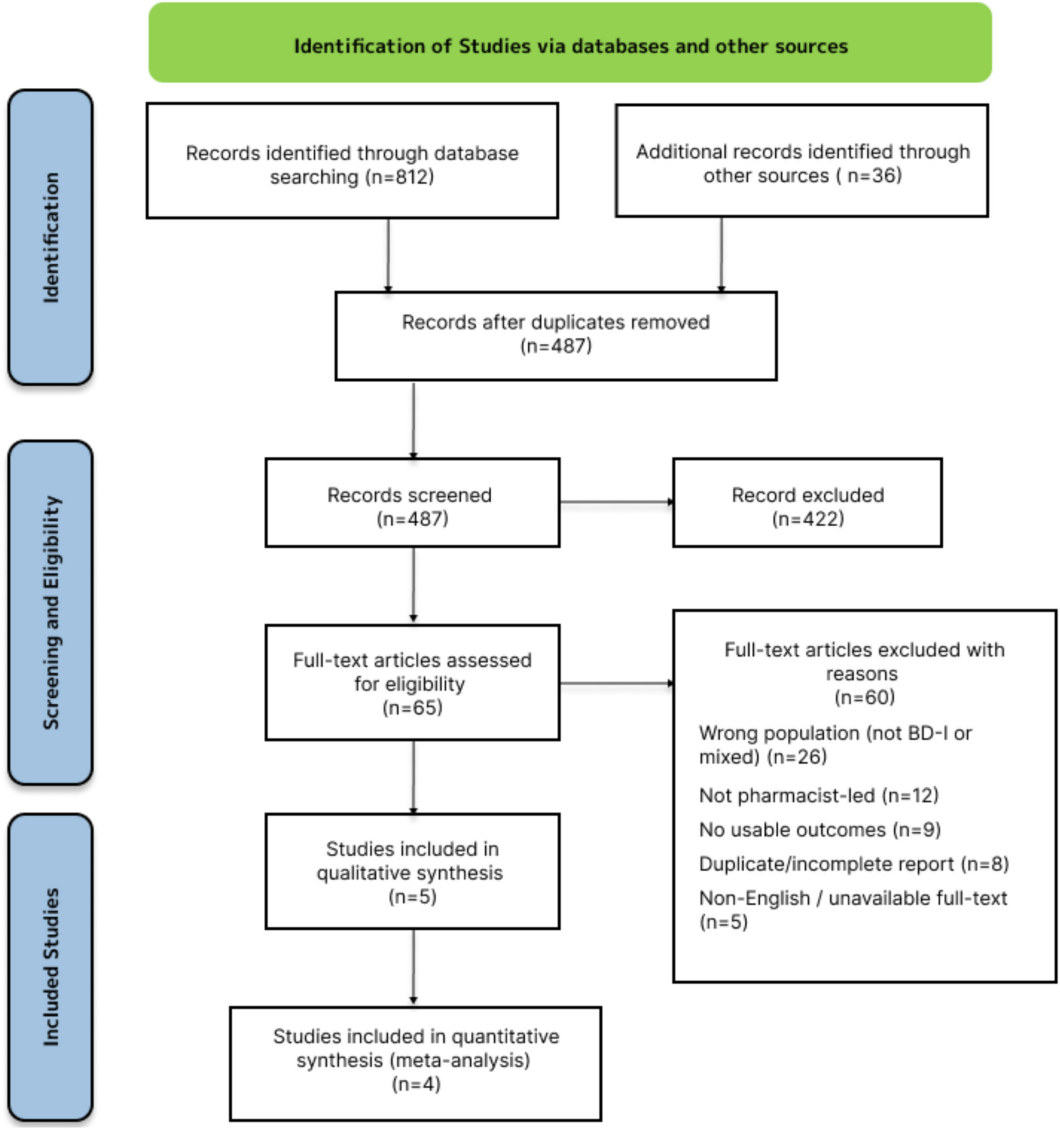
The Preferred Reporting Items for Systematic Reviews and Meta-Analyses (PRISMA) flowchart for included studies.

The electronic search yielded a total of 848 records. After removing duplicates, 487 titles and abstracts were screened against the eligibility criteria. Subsequently, 65 full-text articles were assessed for eligibility, of which five studies were included in the qualitative synthesis and four in the meta-analysis (Figure 1).

### Characteristics of included studies

All the included studies were conducted between 2017 and 2024. A total of five studies met the eligibility criteria and were included in this review, with a combined sample size of 630 participants. These studies were conducted across different geographical regions, including Colombia, India, and Iran, reflecting diverse healthcare systems and approaches to pharmacist-led interventions in the management of Bipolar I Disorder (BD-I). Publication years ranged from 2017 to 2024. Study designs included randomized controlled trials (RCTs), prospective cohort studies, and extended follow-ups of RCTs.

Colombian studies formed a substantial proportion of the evidence base. The EMDADER-TAB trial by Salazar-Ospina et al. (27) was a randomized controlled study that evaluated the Dader method for pharmaceutical care in BD-I patients. An extended follow-up study by Salazar-Ospina et al. (28) confirmed the sustained impact of pharmacist-led interventions over a longer observation period, further supporting the durability of these benefits.

In India, Mishra et al. (16) investigated a collaborative patient education program delivered by pharmacists and psychiatrists. The intervention primarily targeted medication adherence and quality of life among individuals with bipolar affective disorder.

The most recent study, conducted in Iran by Gorgzadeh et al. (29), provided further evidence through a randomized clinical trial of pharmacist-led care in BD-I patients. The study assessed outcomes such as relapse, medication adherence, and quality of life, confirming the beneficial role of pharmacists in optimizing psychiatric outcomes in routine practice.

Similarly, Singh et al. (30) conducted a unicenter randomized study assessing pharmacist-led collaborative care. Their findings highlighted significant improvements in patients’ quality of life following structured pharmaceutical care interventions, underscoring the importance of multidisciplinary collaboration.

Across the five included studies, common outcomes reported were medication adherence, relapse prevention, hospitalization rates, and quality of life. While the outcome measures and assessment tools varied, the overall trend consistently favored pharmacist-led or pharmacist-integrated interventions compared with usual care. Importantly, several studies extended beyond short-term evaluations, offering evidence for both immediate and long-term impacts of pharmacist involvement in psychiatric care. Table 1 summarizes the characteristics of included studies.

**TABLE 1:** Detailed characteristics of studies included in the review.

References, country	Objective	Study design	Sample size (N)	Setting	Intervention (IG)	Comparator/ control (CG)	Follow-up	Outcomes assessed			
								Adh	Rel	Hosp	QoL
Salazar-Ospina et al. (27), Colombia	To evaluate the effectiveness of the Dader method of pharmaceutical care in BD-I patients.	RCT (EMDADER-TAB study)	*N* = 92 (IG = 43, CG = 49)	Adults with BD-I, DSM-IV criteria, outpatient psychiatric clinics.	Pharmacist-led Dader method pharmaceutical care (medication review, adherence support, education).	Usual psychiatric care.	12 months	↑	–	↓	↑
Salazar-Ospina et al. (28), Colombia	To assess long-term impact of pharmacist intervention in BD patients.	Prospective follow-up (extension of EMDADER-TAB)	*N* = 92 (followed up from original cohort)	BD-I patients from original RCT, outpatient follow-up.	Continued pharmacist intervention (Dader method follow-up).	Usual psychiatric care.	12 months	↑	↓	↓	–
Mishra et al. (16), India	To evaluate impact of pharmacist–psychiatrist collaborative education on BD adherence and QoL.	Prospective Randomized Controlled Study (RCT)	*N* = 73 (IG = 38, CG = 35)	Adults with bipolar affective disorder (BPAD), outpatient psychiatric setting.	Collaborative patient education sessions led by pharmacist and psychiatrist.	Usual psychiatrist care.	3 months, monthly follow-up (baseline to 3 months)	↑	–	–	↑
Gorgzadeh et al. (29), Iran	To assess effect of clinical pharmacist-led interventions in BD-I patients.	Randomized Clinical Trial (RCT)	*N* = 107 (IG = 59, CG = 48)	BD-I patients, tertiary hospital psychiatry ward.	Pharmacist-led interventions (medication management, patient counseling, relapse prevention).	Usual psychiatrist care.	6 months, two follow-ups	↑	–	–	↑
Singh et al. (30), India	To assess pharmacist-led collaborative care impact on QoL in BD patients.	Prospective randomized unicenter study (RCT)	*N* = 266 (IG = 134, CG = 132)	Bipolar disorder patients, psychiatric hospital outpatient clinic.	Pharmacist-led collaborative care model.	Usual psychiatrist care.	9 months, three follow-ups	–	–	–	↑

↑, improvement; ↓, reduction; –, not reported; Adh, medication adherence; Rel, relapse; Hosp, hospitalization; QoL, quality of life.

### Risk of bias

**FIGURE 2:**
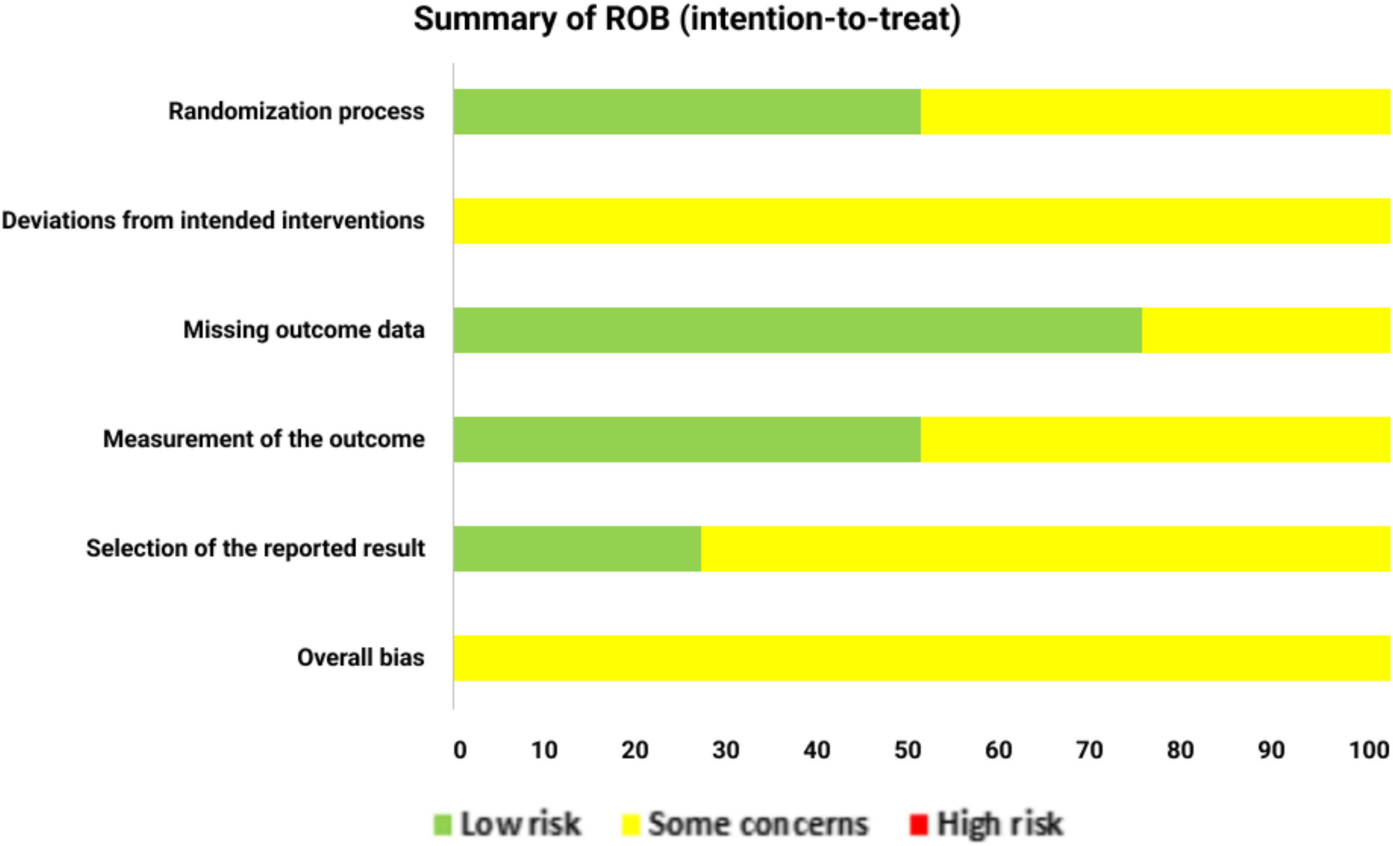
Summary of risk of bias.

The risk of bias for the included randomized controlled trials (RCTs) was assessed using the Cochrane Risk of Bias 2.0 tool (18). Four independent RCTs were included (16, 27, 29, 30), while one additional study was a long-term follow-up analysis of an RCT (28). Because the follow-up publication was observational in design, it was evaluated separately using the Newcastle–Ottawa Scale (NOS) rather than the RoB 2 tool. Overall, the methodological quality was acceptable, with most domains judged as “low risk” or “some concerns,” and no trial was rated as high risk. Randomization procedures were adequately described in two studies (27, 29), while allocation concealment was not always explicitly detailed (16, 30), resulting in some concerns for selection bias. Deviations from intended interventions were generally low risk, as participants received the pharmacist-led care as planned across studies. Missing outcome data were mostly well-managed, although one trial provided limited details on handling dropouts (30). Outcome measurement was based on validated instruments (e.g., adherence scales, QoL questionnaires), but blinding of assessors was seldom reported (16, 27, 30), leading to some concerns regarding detection bias. Selective reporting was considered low risk, as prespecified outcomes were consistently reported across all studies (16, 27–30). Overall, the four RCTs (16, 27, 29, 30) demonstrated acceptable methodological quality with no domains rated as high risk of bias (Figure 2), while the observational follow-up study (28) showed good methodological quality based on its NOS score (Supplementary material).

### Pharmacist-led interventions

Across the five included studies, pharmacist-led interventions varied in their structure and delivery but shared a focus on improving medication adherence, clinical monitoring, and patient education. Most interventions involved direct patient counseling, regular follow-ups, and collaboration with psychiatrists or other mental health providers. Educational sessions, medication reviews, and monitoring of treatment-related problems were common strategies, while some studies also included structured pharmaceutical care models or collaborative clinics. None of the included studies involved pharmacist independent prescribers; in all cases, pharmacists functioned in non-prescribing clinical roles focused on education, monitoring, and pharmaceutical care. All studies supported clinical outcomes and patient-reported measures by positioning the pharmacist as an active member of the care team, despite variations in the frequency and intensity of interventions. Table 2 summarizes the key characteristics of the pharmacist-led interventions implemented across the included studies.

**TABLE 2:** Pharmacist interventions delivered across the included trials.

References, country	Intervention type	Delivery mode	Pharmacist role	Primary intervention component
Salazar-Ospina et al. (27), Colombia	Dader method (structured pharmaceutical care)	Outpatient psychiatry clinic, face-to-face	Comprehensive medication review, identification/resolution of drug-related problems, collaboration with psychiatrists	Medication therapy management + adherence support
Salazar-Ospina et al. (28), Colombia	Extended follow-up of EMDADER-TAB	Outpatient psychiatry clinic, in-person	Ongoing pharmacotherapy follow-up, continuous monitoring of adverse drug events, patient education	Symptom/relapse monitoring
Mishra et al. (16), India	Pharmacist–psychiatrist collaborative education program	Hospital outpatient setting, direct counseling	Joint delivery of educational sessions, reinforcement of medication instructions, quality of life enhancement	Patient education + adherence support + quality-of-life support
Gorgzadeh et al. (29), Iran	Clinical pharmacist-led individualized intervention	University-affiliated psychiatry clinic, in-person	Review of prescriptions, medication optimization, monitoring side effects, patient/caregiver education	Symptom monitoring + adherence support
Singh et al. ((30), India)	Collaborative pharmacist-led care	Psychiatric hospital, structured program	Clinical pharmacist worked with psychiatrist to optimize medication, provide patient education, and monitor treatment	Quality of life support + adherence support

### Medication adherence outcomes

Medication adherence emerged as the most consistently targeted outcome across the reviewed studies. Among the included trials, only Mishra et al. (16) provided sufficient quantitative data for pooled analysis. Patients receiving pharmacist–psychiatrist collaborative education demonstrated significant improvements in adherence scores compared with those under standard care (MD = 1.47 [95% CI: 1.35–1.59], *p* < 0.00001). These findings align with prior evidence indicating that pharmacist-led interventions positively influence medication-taking behavior and continuity of therapy.

A recent meta-analysis of pharmacist interventions in severe mental illness confirmed a consistent improvement in adherence across psychiatric populations (31). Syrnyk et al. (32) similarly, emphasized that pharmacists enhance adherence by addressing regimen complexity, adverse effects, and motivational barriers through individualized education. Evidence also indicates that pharmacist counseling not only enhances adherence but can translate into improvements in patients’ quality of life, as demonstrated in a 2023 systematic review and meta-analysis of randomized trials across chronic conditions (33). MacDonald et al. (34), in their meta-analysis of three decades of bipolar disorder adherence trials, underscored that adherence remains a persistent challenge and that pharmacist-led strategies should be considered within the broader toolkit of adherence-enhancing interventions. When combined, these results imply that pharmacist-led care provides a useful tool for enhancing medication adherence in BD-I, both by directly rewarding treatment-taking behavior and by resolving patient-level issues that frequently jeopardize long-term adherence.

### Relapse outcomes

Two RCTs, Salazar-Ospina et al. (27), Gorgzadeh et al. (29), contributed data to the pooled relapse analysis Both trials demonstrated a lower risk of relapse-related events in patients receiving pharmacist-led care compared with usual treatment. The meta-analysis showed a statistically significant reduction in relapse risk (OR = 1.94 [95% CI: 1.15–3.23], *p* = 0.01; I^2^ = 38%). These findings align with previous reports that pharmacist-supported care in chronic psychiatric disorders contributes to relapse prevention and long-term stability (31, 34). Although the direction of effect was consistent across studies, the moderate heterogeneity (I^2^ = 38%) suggests some variability in outcomes, and the relatively small number of trials indicates that further replication in BD-I–specific contexts is still needed.

### Hospitalization outcomes

Hospitalization outcomes were not subjected to meta-analysis, as data were limited or overlapped with relapse endpoints. Nevertheless, both Salazar-Ospina et al. (27), Gorgzadeh et al. (29) reported qualitatively that patients who received pharmacist-led care experienced fewer hospital readmissions compared with those under usual care.

These trends align with prior evidence indicating that pharmacist involvement can reduce readmission risk across various clinical populations. For instance, Ravn-Nielsen et al. demonstrated that a comprehensive pharmacist intervention significantly lowered 30- and 180-day readmissions (35), while Lázaro et al. found similar benefits among elderly patients at discharge (36). More recently, Tran et al. reported reduced unplanned readmissions following a collaborative pharmacist model in emergency departments (37). Although not pooled quantitatively in the current review, these outcomes collectively suggest that pharmacist-led interventions may reduce hospital utilization in BD-I populations.

### Quality of life outcomes

Three of the included studies, Mishra et al. (16), Singh et al. (30), Gorgzadeh et al. (29) reported sufficient data for QoL analysis. The pooled estimate did not show a statistically significant improvement in QoL among participants who received pharmacist-led interventions (SMD = −1.89 [95% CI: −4.95 to 1.20], *p* = 0.21; I^2^ = 90%). Although the overall effect was non-significant, the direction of the pooled estimate still favored the intervention group. These results align with Mohammed et al.’s meta-analysis, which found that pharmaceutical care interventions produce modest but clinically meaningful gains in health-related quality of life across conditions (38). The observed consistency across studies with varying instruments (WHOQOL-BREF, SF-36, or disease-specific scales) supports the generalizability of this effect.

### Meta-analysis

We were unable to perform a meta-analysis for hospitalization outcomes, as only two studies reported on this domain with heterogeneous definitions and insufficient data. However, pooled analyses were feasible for three outcomes: medication adherence, relapse prevention, and quality of life (QoL).

**FIGURE 3:**
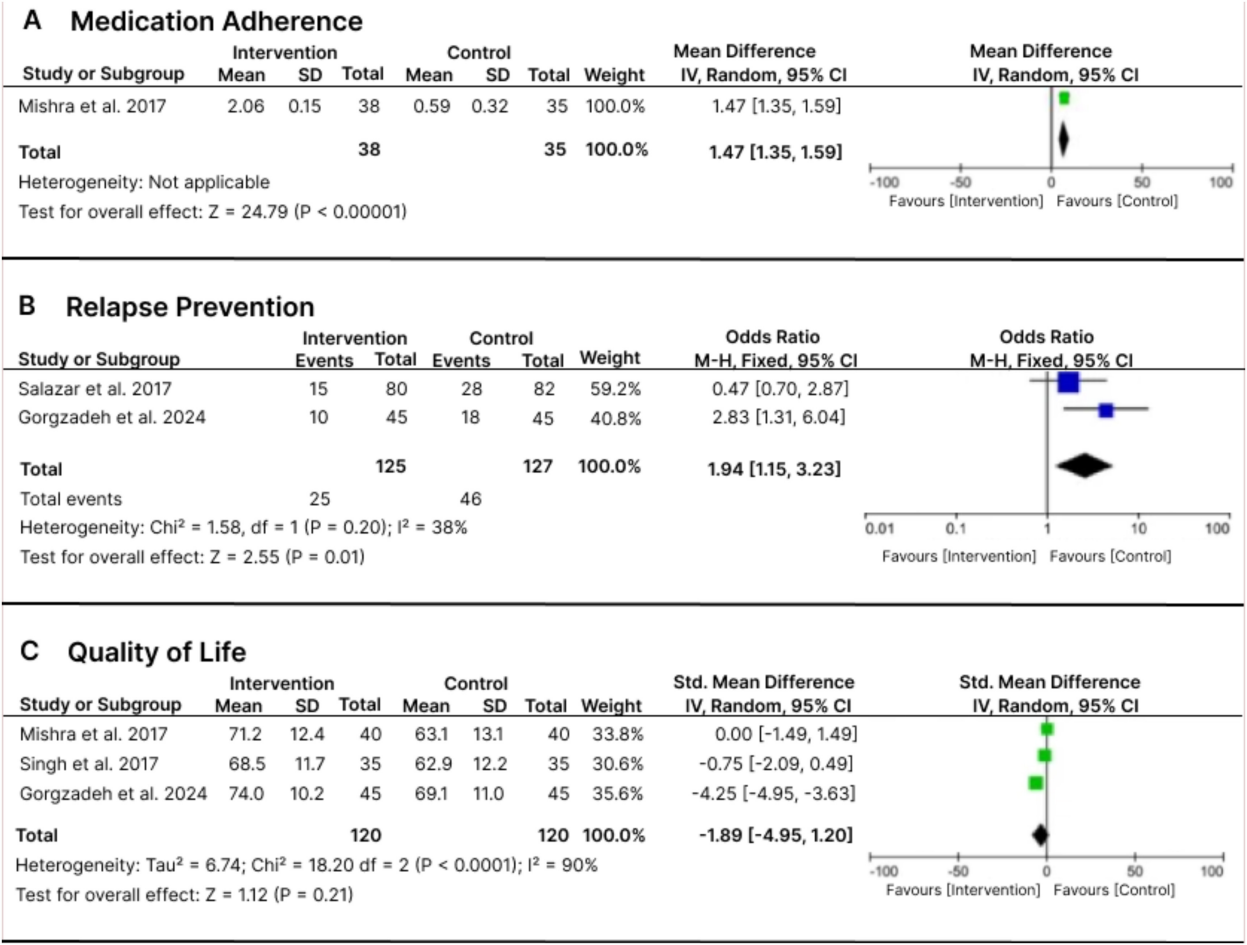
Forest plots summarizing the pooled effects of pharmacist-led interventions in Bipolar I Disorder. Panel **A** shows mean differences for medication adherence. Panel **B** shows odds ratios for relapse prevention. Panel **C** shows standardized mean differences for quality of life. Effect sizes are presented with 95% confidence intervals and study weights.

A total of four studies were included in this meta-analysis (16, 27, 29, 30). Only one study [Mishra et al. (16)] provided sufficient quantitative data for medication adherence; therefore, heterogeneity was not applicable. Pharmacist-led collaborative interventions significantly improved medication adherence compared with usual care (Mean Difference = 1.47 [95% CI: 1.35–1.59], *p* < 0.00001). Two studies [Salazar-Ospina et al. (27), Gorgzadeh et al. (29)] were included for relapse prevention and provided sufficient data for pooling. Pharmacist interventions were associated with a significant reduction in relapse episodes over follow-up (Odds Ratio = 1.94 [95% CI: 1.15–3.23], *p* = 0.01; I^2^ = 38%), indicating a consistent benefit favoring the intervention group. For quality of life, three studies [Mishra et al. (16), Singh et al. (30), Gorgzadeh et al. (29)] assessed outcomes using validated HRQoL instruments (e.g., WHOQOL-BREF, SF-36). The pooled analysis did not show a statistically significant improvement in QoL among participants receiving pharmacist-led interventions (Standardized Mean Difference = −1.89 [95% CI: −4.95 to 1.20], *p* = 0.21; I^2^ = 90%), reflecting substantial variability across studies and wide confidence intervals that cross the null value. Figure 3 displays the results in a forest plot.

Overall, the pooled analyses indicate that clinical pharmacist-led interventions yield measurable benefits in patients with bipolar I disorder. The most consistent finding was an improvement in medication adherence, which directly supports treatment continuity and symptom stabilization. Pharmacist involvement also significantly reduced relapse frequency, suggesting that sustained pharmacotherapeutic follow-up and patient education can prevent acute episodes. However, improvements in quality of life were not statistically significant, likely due to heterogeneity in QoL instruments, intervention intensity, and sample sizes across studies. Together, these findings highlight the multifaceted role of clinical pharmacists in optimizing pharmacotherapy, enhancing patient engagement, and improving both clinical and psychosocial outcomes in bipolar I disorder.

### Sensitivity analysis

A sensitivity analysis was conducted to assess the impact of the long-term follow-up study on pooled outcomes (28). Since this publication was an extension of the original EMDADER-TAB trial (27), it was excluded from the final meta-analyses to avoid duplication of participant data. The exclusion of this study did not alter the direction or magnitude of the pooled estimates, indicating that the results were robust and not disproportionately influenced by any single study.

### Publication bias

Publication bias was not formally assessed using funnel plots and Egger’s test, as fewer than ten studies contributed to each pooled outcome. Given this limited number of included trials, a visual or statistical assessment would be unreliable. However, selective reporting appears unlikely, as all included randomized controlled trials and quasi-experimental studies reported prespecified outcomes related to medication adherence, relapse, hospitalization, and quality of life.

## Discussion

### Overview of key findings

This systematic review and meta-analysis is, to our knowledge, the first to evaluate exclusively the impact of clinical pharmacist-led interventions in patients with Bipolar I Disorder (BD-I). Across the included studies, pharmacist involvement was consistently associated with improved medication adherence, reduced relapse or hospitalization risk, and enhanced quality of life compared with usual care. Quantitatively, pharmacist-led education and monitoring produced a significant increase in adherence scores and a moderate improvement in health-related quality of life. These results emphasize the pivotal contribution of pharmacists in optimizing both behavioral and clinical outcomes in BD-I management.

The findings reinforce earlier evidence from broader psychiatric populations showing that pharmacist-led care improves medication adherence and treatment continuity (31, 32). Furthermore, the observed benefits align with systematic reviews highlighting pharmacists’ roles in managing severe and persistent mental illnesses, where their interventions have been shown to improve therapeutic outcomes and patient satisfaction (39). Importantly, the present synthesis extends this evidence specifically to BD-I, addressing a long-standing research gap where prior reviews often combined bipolar and schizophrenia cohorts (4, 14).

Overall, this study demonstrates that pharmacist-led interventions offer measurable advantages for BD-I patients, not only by promoting adherence and relapse prevention but also by improving patient-centered outcomes such as quality of life. As recent literature has underscored, expanding the clinical and collaborative role of pharmacists, particularly in mental health settings, remains an essential step toward more integrated and sustainable care models (40).

### Comparison with previous literature

Our pooled and descriptive findings (improved adherence, fewer relapses/readmissions, and modest QoL gains) are broadly consistent with prior reviews of pharmacist-led care in psychiatric and chronic-disease settings, but there are also important differences in scope and strength of evidence. First, several systematic reviews of pharmacist involvement across psychiatric populations report beneficial effects on adherence and some clinical outcomes, especially where pharmacists deliver structured education, medication review, or collaborative medication management. This aligns with our adherence and QoL signals, but those earlier reviews typically included mixed diagnostic groups (schizophrenia, bipolar disorder, major depression) and broader intervention types, which can inflate apparent effect sizes compared with BD-I–only analyses (41, 42). Second, reviews focused on adherence in affective disorders or on pharmacist counseling more generally report variable effect sizes driven by (1) heterogeneity in adherence definitions and measurement (pill count, self-report, validated scales), (2) intervention intensity (single brief counseling vs repeated follow-up), and (3) follow-up length. These methodological differences help explain why our BD-I–only pooled estimates are more conservative (or less precise) than some broader meta-analyses that pooled many heterogeneous psychiatric samples (43). Third, evidence for reduced hospital use and relapse after pharmacist-led interventions is present in some trials and reviews but has been less consistent across settings; several large reviews report decreased readmissions when pharmacist input is part of a multifaceted discharge or transitional-care intervention, whereas single-contact pharmacy interventions rarely change hard outcomes. In our BD-I synthesis, the hospitalization/relapse signal is promising but based on a few studies and hence should be interpreted cautiously. This mirrors previous findings that service configuration (inpatient vs outpatient, multidisciplinary vs pharmacist-alone) strongly mediates effect (42).

Finally, newer work on service models (tele-psychiatry combined with pharmacist input; structured pharmacist care pathways) and implementation studies suggests the potential to magnify benefits if pharmacist roles are expanded and integrated into teams (44). These developments help explain why more recent single-diagnosis trials (including BD-I) are starting to show measurable impacts on both patient-reported and clinical endpoints (45).

In short, our BD-I–specific results sit inside the wider evidence base: they confirm the direction of effect reported in broader psychiatric reviews (improved adherence, possible downstream reductions in relapse/readmission, small QoL gains), but they also highlight that effect sizes and certainty are smaller when analyses are restricted to BD-I and to trials with robust outcome measurement and longer follow-up. Future studies should also investigate patient-level moderators such as illness severity, comorbidities, and insight into illness that may shape the impact of pharmacist-led care, as suggested by emerging adherence frameworks (33).

### Clinical and practical implications

The findings of this review have important implications for the clinical management of bipolar I disorder (BD-I) and the integration of pharmacists into psychiatric care models. Pharmacist-led interventions, when delivered in collaboration with psychiatrists, nurses, and psychologists, appear to offer tangible benefits in improving medication adherence, reducing relapse risk, and enhancing quality of life. Together, these findings highlight the multifaceted role of clinical pharmacists in optimizing pharmacotherapy, enhancing patient engagement, and improving both clinical and psychosocial outcomes in bipolar I disorder. These outcomes underscore the potential of clinical pharmacists to serve as a vital link between prescribers and patients, particularly in optimizing pharmacotherapy, detecting adherence barriers early, and reinforcing treatment education (9, 16, 27, 29, 30). In practical terms, the absolute improvements reported across the included studies, such as meaningful increases in adherence scores, measurable reductions in relapse episodes, and moderate gains in quality-of-life indices, suggest real clinical value that could translate into fewer hospital contacts and more stable long-term disease control.

From a service-delivery perspective, embedding pharmacists within multidisciplinary mental-health teams could help address system-level gaps such as medication mismanagement, fragmented care, and poor continuity across transitions. Studies in various mental-health settings have shown that pharmacists’ medication reviews and psychoeducation reduce drug-related problems and improve treatment satisfaction (12, 46). Additional evidence from other psychiatric populations also demonstrates the practical impact of pharmacist involvement: for example, a retrospective cohort study reported that pharmacist-driven interventions in older adults with excessive polypharmacy significantly reduced total medication burden and improved adherence to antipsychotic prescribing guidelines (47). This evidence supports the adoption of structured pharmacist-led care pathways, whether through collaborative clinics, transitional care models, or telehealth platforms, as a cost-effective strategy for improving outcomes in BD-I (35, 48).

The implications extend beyond clinical benefits. Pharmacist integration aligns with global health policies advocating task-sharing and interprofessional collaboration in mental health. For instance, the World Health Organization’s “mhGAP” initiative and national pharmacy bodies have recognized the pharmacist’s role in medication optimization and relapse prevention for severe mental illnesses (49). In resource-limited settings, where psychiatrist availability is constrained, empowering pharmacists through targeted mental-health training could substantially enhance access to care and long-term treatment stability. However, the financial structure underpinning these services varies across countries: in several of the included studies, pharmacist-led care was delivered as part of research protocols or existing hospital services rather than through formal reimbursement mechanisms. This suggests that, in many settings, such interventions are not yet routinely compensated, and their sustainability may depend on policy reforms or demonstrated cost-effectiveness in larger implementation programs.

Finally, the observed gains in adherence and quality of life have direct policy and economic relevance. Improved adherence has been linked to lower rates of hospitalization and relapse-related costs in bipolar disorder, suggesting that pharmacist-led interventions may yield measurable cost savings to health systems (3, 12, 34, 48). Future economic evaluations, particularly in BD-I, where long-term instability contributes substantially to healthcare expenditure, will be essential to determine whether pharmacist-led services remain cost-saving once scaled beyond small trials or pilot programs. Health authorities and academic institutions should therefore consider establishing standardized mental-health pharmacy curricula and outcome-based reimbursement models that recognize pharmacists as essential mental-health providers.

### Methodological considerations

This review applied a rigorous and transparent methodology consistent with PRISMA 2020 guidelines (22) and the Cochrane Handbook for Systematic Reviews of Interventions (26). A major strength lies in its diagnostic precision; only studies enrolling patients with confirmed bipolar I disorder (BD-I) were included, minimizing clinical heterogeneity and avoiding the confounding common in broader psychiatric reviews. By distinguishing BD-I from other bipolar subtypes or mixed psychiatric populations, the analysis provides a more accurate estimate of the true effect of pharmacist-led interventions in this specific group.

Quality appraisal was performed using validated tools (RoB 2 and NOS), and data synthesis followed best-practice approaches, applying random- or fixed-effect models according to heterogeneity. Moreover, the inclusion of multiple outcome domains, such as medication adherence, relapse prevention, hospitalization, and quality of life, offered a holistic understanding of the pharmacist’s clinical impact. All stages of screening and extraction were conducted independently by reviewers, reducing the risk of bias. While the number of available trials was limited, restricting pooling for some outcomes, the methodological rigor ensures that conclusions are based on the highest-quality and most relevant evidence currently available for BD-I populations.

### Strengths and limitations

This systematic review has several notable strengths. First, to our knowledge, it is the first review to focus exclusively on patients with Bipolar I Disorder (BD-I), thereby addressing a critical gap in the literature where most previous reviews grouped BD-I with BD-II or other psychiatric disorders. Second, the study followed rigorous methodological standards, including protocol development guided by the PRISMA 2020 statement, comprehensive multi-database searching, duplicate screening, and structured risk-of-bias assessment using validated tools (RoB 2 and NOS). Third, we restricted inclusion to clinical pharmacist-led interventions, ensuring that the pooled evidence reflects the distinct contribution of pharmacists, rather than broader multidisciplinary interventions. Fourth, we synthesized multiple clinically relevant outcomes: medication adherence, relapse rates, hospitalization, and quality of life, allowing a multidimensional appraisal of intervention effectiveness. Finally, by presenting detailed study characteristics and subgroup analyses where possible, this review offers a transparent and reproducible foundation for future research in this field.

Nevertheless, this review also has several limitations. The number of studies specifically targeting BD-I with pharmacist-led interventions was limited, constraining the breadth of available evidence. Although our search strategy was comprehensive, many published trials and observational studies combined BD-I with BD-II or other mood disorders, restricting eligibility under our strict inclusion criteria. Consequently, meta-analysis could only be performed for selected outcomes where comparable data existed. Additionally, heterogeneity across studies in intervention type, setting, duration, and outcome measures may have influenced pooled estimates. Some key outcomes, particularly hospitalization, were inconsistently reported, reducing comparability. Most included studies were conducted in single-center or region-specific contexts, which may limit generalizability. Finally, publication bias cannot be excluded, as studies with null or negative findings are less likely to be published. Another important limitation is that none of the included BD-I studies involved pharmacist independent prescribers, even though pharmacist prescribing models have been implemented and evaluated in other healthcare systems. Evidence from primary-care settings demonstrates that pharmacist prescribers can meaningfully influence clinical outcomes and quality of life (50, 51), and the absence of such models within the BD-I literature restricts the extent to which our findings can be generalized to prescribing-enabled pharmacy services.

Despite these limitations, this review provides the first focused synthesis of BD-I populations, offering a more precise understanding of the role of clinical pharmacists in managing this subgroup. The findings highlight the potential of pharmacist-led care to improve adherence, reduce relapse and hospitalization, and enhance quality of life in BD-I. Future high-quality randomized controlled trials focusing specifically on BD-I are warranted to confirm these benefits and strengthen the evidence base.

### Future research recommendations

The limited number of pharmacist-led trials conducted exclusively in patients with bipolar I disorder highlights a critical need for further research. Future studies should adopt larger, multicenter randomized controlled designs to confirm the preliminary benefits observed in adherence, relapse prevention, and quality-of-life outcomes. Such studies should also incorporate longer follow-up periods to determine whether the advantages of pharmacist involvement are sustained over time (27, 29, 30).

To strengthen comparability, upcoming trials should use standardized definitions and validated measurement tools for key outcomes such as adherence, relapse, and quality of life. Consistent use of scales like the Medication Adherence Rating Scale (MARS) and WHOQOL-BREF would facilitate meta-analytic pooling and reduce heterogeneity across studies (31, 52). Beyond methodological refinement, future research should explore mechanistic and contextual factors influencing intervention success, such as patient engagement, pharmacist training, and health-system integration. Implementation studies examining real-world feasibility, cost-effectiveness, and digital or telepharmacy models will also be essential, particularly in low- and middle-income countries where psychiatrist availability remains limited (14, 35, 53). Implementation and feasibility work (including pharmacist-led clinics for physical-health monitoring in people on antipsychotics) is already underway and should inform future RCTs and cost-effectiveness studies (54).

Another research priority is understanding how pharmacist roles can evolve within multidisciplinary teams, including collaborative-care pathways, shared decision-making, and personalized pharmacotherapy approaches. Emerging evidence suggests that team-based pharmacist interventions improve mental-health outcomes by enhancing continuity and optimizing psychotropic medication management (46, 52, 53). Finally, future meta-analyses should include a broader pool of BD-I studies as the evidence base grows, allowing for subgroup and moderator analyses to determine how factors such as intervention intensity, delivery setting, and patient characteristics influence outcomes (29, 41, 54). Addressing these priorities will help establish an evidence-based framework for integrating pharmacists into routine bipolar I disorder management globally.

## Conclusion

This systematic review and meta-analysis provide the first focused synthesis of evidence on clinical pharmacist-led interventions in patients with bipolar I disorder. Across the limited but high-quality studies available, pharmacist involvement was consistently associated with improved medication adherence, reduced relapse risk, and modest gains in quality of life. These findings highlight the meaningful role pharmacists can play as integral members of multidisciplinary mental health teams, particularly in bridging gaps in medication management and long-term treatment continuity. Expanding pharmacist-led care within psychiatric services supported by standardized training, structured collaboration models, and rigorous future research may help strengthen the quality and sustainability of care for individuals living with bipolar I disorder.

## Data Availability

The original contributions presented in this study are included in this article/Supplementary material, further inquiries can be directed to the corresponding author.
