# Supplementary material for: Clinical pharmacist-led interventions and their impact on outcomes in patients with bipolar I disorder: a systematic review and meta-analysis
Source: Front Med (Lausanne). 2025 Dec 12;12:1725491. doi: 10.3389/fmed.2025.1725491 (PMC12741269; doi:10.3389/fmed.2025.1725491)
Supplement: Supplementary file 1 [file Supplementary_file_1.docx]

**Appendix A — Electronic Database Searches**

**PubMed/MEDLINE**

#1 "Bipolar I Disorder"[Mesh] OR "bipolar I disorder"[tiab] OR "bipolar affective disorder"[tiab] OR "manic depression"[tiab] OR "manic-depressive illness"[tiab]

#2 "Pharmacists"[Mesh] OR pharmacist*[tiab] OR "clinical pharmacist"[tiab] OR "pharmacist-led"[tiab] OR "pharmacist-managed"[tiab] OR "pharmacist intervention"[tiab] OR "pharmaceutical care"[tiab] OR "medication therapy management"[tiab] OR "collaborative care"[tiab]

#3 "Medication Adherence"[Mesh] OR "medication adherence"[tiab] OR "treatment adherence"[tiab] OR "treatment compliance"[tiab]

#4 "Relapse"[Mesh] OR relapse*[tiab] OR "recurrence"[tiab] OR "readmission"[tiab] OR "rehospitalization"[tiab] OR "hospital readmission"[tiab]

#5 "Quality of Life"[Mesh] OR "quality of life"[tiab] OR QoL[tiab] OR "health-related quality of life"[tiab]

#6 #1 AND #2 AND (#3 OR #4 OR #5)

Limits: Humans, English, Publication years 2000–2025

**Embase (via Elsevier)**

#1 'bipolar i disorder'/exp OR 'bipolar i disorder':ti,ab OR 'bipolar affective disorder':ti,ab OR 'manic depression':ti,ab

#2 'pharmacist'/exp OR pharmacist*:ti,ab OR 'clinical pharmacist':ti,ab OR 'pharmaceutical care':ti,ab OR 'medication therapy management':ti,ab OR 'pharmacist intervention':ti,ab OR 'collaborative care':ti,ab

#3 'medication compliance'/exp OR 'medication adherence':ti,ab OR 'treatment compliance':ti,ab OR 'treatment adherence':ti,ab

#4 'relapse'/exp OR relapse*:ti,ab OR 'readmission':ti,ab OR 'hospitalization':ti,ab OR 'rehospitalization':ti,ab

#5 'quality of life'/exp OR 'quality of life':ti,ab OR QoL:ti,ab OR 'health-related quality of life':ti,ab

#6 #1 AND #2 AND (#3 OR #4 OR #5)

Filters: Human, English, Year = 2000–2025

**PsycINFO**

#1 DE "Bipolar Disorder" OR "bipolar I disorder" OR "bipolar affective disorder" OR "manic depression"

#2 DE "Pharmacists" OR pharmacist* OR "clinical pharmacist" OR "pharmacist intervention" OR "pharmacist-led" OR "pharmaceutical care" OR "collaborative care" OR "medication therapy management"

#3 DE "Medication Compliance" OR "medication adherence" OR "treatment adherence" OR "treatment compliance"

#4 DE "Relapse" OR relapse* OR "readmission" OR "hospital readmission"

#5 DE "Quality of Life" OR "health-related quality of life" OR QoL

#6 #1 AND #2 AND (#3 OR #4 OR #5)

Limits: Human, English, 2000–2025

**Scopus, Web of Science, and Cochrane Library**

(TITLE-ABS-KEY("bipolar I disorder" OR "bipolar affective disorder" OR "manic depression"))

AND (TITLE-ABS-KEY(pharmacist* OR "clinical pharmacist" OR "pharmacist-led" OR "pharmaceutical care" OR "medication therapy management" OR "collaborative care"))

AND (TITLE-ABS-KEY("medication adherence" OR "treatment adherence" OR "relapse" OR "readmission" OR "quality of life" OR QoL))

AND (LIMIT-TO(LANGUAGE, "English"))

AND (PUBYEAR > 1999 AND PUBYEAR < 2026)

**Appendix B — Risk of bias (RoB 2.0) — per-RCT domain judgments**

*(Four randomized trials; judgments made by two independent reviewers using RoB 2.0)*

| **Study (Author, Year)** | **Randomization process** | **Deviations from intended interventions** | **Missing outcome data** | **Measurement of outcome** | **Selection of reported result** | **Overall bias** |
| --- | --- | --- | --- | --- | --- | --- |
| Salazar-Ospina et al., 2017 | Low risk | Some concerns | Low risk | Some concerns | Some concerns | Some concerns |
| Mishra et al., 2017 | Low risk | Some concerns | Low risk | Low risk | Low risk | Some concerns |
| Gorgzadeh et al., 2024 | Some concerns | Some concerns | Low risk | Low risk | Some concerns | Some concerns |
| Singh et al., 2017 | Some concerns | Some concerns | Some concerns | Some concerns | Some concerns | Some concerns |
| **Note:** Overall bias was judged in accordance with the Cochrane Risk of Bias 2.0 guidance, which stipulates that the overall judgment reflects the highest level of concern identified across any of the five domains rather than the majority assessment. Thus, even if most domains were rated as Low risk, the overall bias was categorized as Some concerns if any single domain presented potential bias that could influence the outcome. | | | | | | |

**Non-randomized follow-up (NOS)**

| **Study** | **Selection (max 4★)** | **Comparability (max 2★)** | **Outcome/Exposure Assessment (max 3★)** | **Total Score (max 9★)** | **Quality Judgment** |
| --- | --- | --- | --- | --- | --- |
| Salazar-Ospina et al. (2020) | ★★★☆ | ★★ | ★★☆ | 7/9 | Low risk of bias |
